# Complete Genome Sequence of Herpes Simplex Virus 2 Strain 333

**DOI:** 10.1128/MRA.00870-18

**Published:** 2018-09-06

**Authors:** Alberto Domingo López-Muñoz, Alberto Rastrojo, Antonio Alcamí

**Affiliations:** a Centro de Biología Molecular Severo Ochoa (Consejo Superior de Investigaciones Científicas and Universidad Autónoma de Madrid), Madrid, Spain; University of Maryland School of Medicine

## Abstract

Herpes simplex virus 2, or human herpesvirus 2, is a ubiquitous human pathogen that causes genital ulcerations and establishes latency in sacral root ganglia. We fully sequenced and manually curated the viral genome sequence of herpes simplex virus 2, strain 333 using Pacific Biosciences and Illumina sequencing technologies.

## ANNOUNCEMENT

Herpes simplex virus 2 (HSV-2) is commonly found as the causative agent of genital tract infections. This member of the *Alphaherpesvirinae* subfamily within the *Herpesviridae* virus family is a highly prevalent human pathogen which infects through the genital mucosa, remains latent in dorsal-sacral root ganglia, and produces a lifelong infection. The worldwide prevalence rates are close to 20 to 25%, depending on the country and economic status (1, 2). HSV-2 strain 333, originally isolated from a primary genital lesion from a patient at the Baylor College of Medicine (Houston, TX, USA), has been used by many laboratories as a highly neurovirulent strain in adult mice (3, 4). A partial consensus genome sequence of this strain (GenBank accession no. KP192856) was described as having 153,333 bp, based on alignment of Illumina reads against the HG52 reference genome (4).

A single viral clone was passaged 3 times in cell culture with plaque isolation. Then, that purified clone was amplified by infecting 5 subconfluent P150-cm^2^ culture dishes of Vero cells at 0.1 multiplicity of infection (MOI). After 2 h, the viral inoculum was removed, and the cells were overlaid with 20 ml fresh Dulbecco’s modified Eagle’s medium complete (DMEMc) with 2% fetal bovine serum (FBS). Then, 48 h postinfection, cells and supernatant were collected and concentrated with low-speed centrifugation. Viral particles were purified in a 36% sucrose cushion with ultracentrifugation at 20,000 × *g* over 1 h. Then, the eluted pellet was treated with DNase I to remove cellular DNA. Viral genomic DNA was purified by a proteinase K-SDS treatment followed by phenol-chloroform extraction.

A 20-kb PacBio library was prepared using the BluePippin size selection protocol (7-kb cutoff). This library was sequenced on a PacBio RS II instrument using P6-C4 chemistry with a 360-min movie time, in one single-molecule real-time (SMRT) cell, at the Norwegian Sequencing Centre, University of Oslo (Norway), and we obtained 80,266 reads. Reads longer than 12 kb were *de novo* assembled with HGAP v.3 (5), which gave 1 contig of 155,500 bp with an average coverage of 1,409×. Viral DNA was also sequenced using Illumina technology. This library was prepared with the NEBNext kit and sequenced on a MiSeq device at MicrobesNG, University of Birmingham (UK), which gave 211,368 paired-end reads (2 × 250 bp). Reads were filtered (Prinseq v.1.2) and mapped (Bowtie 2 v.2.3.4.1) as previously described (6) against this PacBio assembly.

The HG52 reference strain was used to annotate strain 333 using BLAST alignments followed by manual curation. *UL32* and *UL36* genes were truncated relative to strain HG52 due to the presence of 2 single-nucleotide deletions and 1 single-nucleotide deletion, respectively. Using Illumina read mapping, those indels contained in the annotated coding DNA sequence (CDS) were corrected manually in the PacBio contig. The final assembly (155,503 bp), was 828 bp longer than the strain HG52 reference sequence (GenBank accession no. NC001798) and showed small differences in both ends but shared 99% genome identity. Despite the use of long reads for the assembly, the “a” sequences at both ends were not fully resolved, and only the central one was complete. Although repeated regions are considered equal, small differences were observed between them.

### Data availability.

The viral genome sequence of HSV-2 strain 333 was deposited in ENA/GenBank under the accession no. LS480640.
